# Septin 9 promoter region methylation in free circulating DNA—potential role in noninvasive diagnosis of lung cancer: preliminary report

**DOI:** 10.1007/s12032-014-0917-4

**Published:** 2014-03-16

**Authors:** Tomasz Powrózek, Paweł Krawczyk, Tomasz Kucharczyk, Janusz Milanowski

**Affiliations:** 1 Department of Pneumonology, Oncology and Allergology, Medical University of Lublin, Jaczewskiego 8, 20-954 Lublin, Poland; 2 Postgraduate School of Molecular Medicine, Warsaw Medical University, Warsaw, Poland; 3 Institute of Agricultural Medicine of Lublin, Lublin, Poland

**Keywords:** Lung cancer, DNA methylation, Septin 9, Free circulating DNA

## Abstract

Currently, there are no sensitive diagnostic tests that could allow early detection of lung cancer. Among some cancer patients, epigenetic changes in the nature of methylation of different gene promoter regions are observed, which affect expression of suppressor genes such as septin 9 (*SEPT9*). Due to the ability of detecting these changes in free circulating DNA in peripheral blood, such genes may become ideal markers in early and noninvasive diagnostics of cancer. Methylation of *SEPT9* promoter region in plasma DNA is observed frequently in colorectal cancer patients. The aim of the study was to define the frequency of *SEPT9* promoter methylation in lung cancer patients and evaluation of usefulness of this marker in early diagnostic of lung cancer. Plasma samples were obtained from 70 untreated patients with different lung cancer pathological diagnosis and disease stage and from 100 healthy individuals. DNA was isolated from peripheral blood plasma and was then subjected to bisulfitation, purification and elution using Abbott mSEPT9 Detection Kit. Methylation level was assessed by real-time PCR with the use of specific *SEPT9* promoter methylation probe. Each sample was assayed in the presence of positive and negative control. *SEPT9* promoter methylation was detected in 31 (44.3 % of the whole studied group) of lung cancer patients finding the result positive when methylation was detected in 1 out of 3 repetitions of each test sample determinations. The marker was present in patients with different pathological diagnosis and disease stage. Analysis of *SEPT9* promoter region methylation may be useful in early diagnosis of lung cancer.

## Introduction

Lung cancer is the most frequent malignancy in the world and is a major cause of cancer deaths in developed countries. Unfortunately, in many cases, the disease is diagnosed too late, in an inoperable stage III or IV, and the 5-year survival rate usually does not exceed 10 % [1, 3]. Despite developments in understanding lung cancer biology and the advances of molecular biology techniques, which allow to use personalized molecularly targeted treatment based on predictive markers (mutations, gene rearrangements), there is still lack of sensitive and specific tests to detect early neoplastic lesions. Currently, in the world, there are no set criteria of screening for the early detection of lung cancer, although attempts are being made to use computed tomography (CT) in the group of intensive smokers. Although CT and positron emission tomography (PET) allow to visualize early neoplastic changes in lungs, it is an expensive examination and the number of patients in the program is small. In addition, such examinations may pose diagnostic problems, providing false-positive results and revealing high percentage of nodules of indeterminate and often non-benign nature [1–3]. The greatest hopes for the early diagnosis of lung cancer are associated with the search for suitably sensitive and specific biological markers detectable by molecular biology techniques in peripheral blood.

In some cancer patients, epigenetic changes in the nature of methylation of different gene promoter regions is observed, which affects their expression [4]. Such phenomena may be detected in free circulating DNA (fc-DNA) of peripheral blood. One of the recently discovered epigenetic changes is methylation of septin 9 (*SEPT9*) gene. It is a suppressor gene whose expression disorders are probably one of the causes of few cancer types development. Decrease in expression of this gene is most often connected with methylation of its promoter region, which induces cancer cell proliferation and migration. It results in an acceleration of tumor growth and facilitates creation of distant metastases. Decreased expression of *SEPT9* gene was observed in colon, prostate and breast cancers. Probably, the decreased expression of *SEPT9* is also found in tumors of ovary, pancreas, lung, kidney, liver, thyroid, and esophagus [5]. Increased promoter methylation of *SEPT9* in fc-DNA of peripheral blood plasma appears to be particularly common in colorectal cancer patients, and the assessment of methylation in this type of malignancy is nowadays possible with commercial CE-IVD (in vitro diagnostic) test. However, there are no reports about the frequency of this phenomenon in lung cancer patients.

The aim of the study was to assess the frequency of *SEPT9* promoter methylation in patients with various pathological types and stages of lung cancer, as well as a pilot evaluation of usefulness of this marker in early diagnostic of the disease.

## Materials and methods

Evaluation of *SEPT9* promoter methylation in fc-DNA was carried out in 70 lung cancer patients (45 men and 25 women, median age 65 years) and 100 healthy individuals (62 men and 38 women, median age 58 years) of Caucasian origin. Control group and examined group were matched in term of gender, age and smoking status. Based on pathologic diagnosis, small cell lung cancer (SCLC) was diagnosed in 23 patients (33 % of the studied group) and non-small cell lung cancer (NSCLC) was diagnosed in 47 patients (67 % of the studied group). None of the patients had radical surgery nor chemo- or radiotherapy. Detailed characteristic of patients is shown in Table 1.

**Table 1:** Frequency of SEPT9 promoter methylation in patients with different pathological diagnosis and different disease stage

	mS9 (−) negative (0/3)	mS9 (+) positive			
		All mS9 (+)	1/3	2/3	3/3
Median age ± SD (years)	65 ± 9	64 ± 7	64 ± 10	67 ± 7	60 ± 5
All patients (*n* = 70)	39 (56 %)	31 (44 %)	7 (10 %)	8 (11 %)	16 (23 %)
Sex					
Women (*n* = 25; 36 %)	15 (60 %)	10 (40 %)	4 (16 %)	–	6 (24 %)
Men (*n* = 45; 64 %)	24 (53 %)	21 (47 %)	3 (9 %)	8 (18 %)	10 (22 %)
Pathological diagnosis					
NSCL patients (*n* = 47; 67 %)	22 (47 %)	25 (53 %)	3 (6 %)	7 (15 %)	15 (32 %)
SCLC patients (*n* = 23; 33 %)	17 (74 %)	6 (26 %)	4 (17 %)	1 (4.5 %)	1 (4.5 %)
Adenocarcinoma (*n* = 20; 29 %)	9 (45 %)	11 (55 %)	3 (15 %)	1 (5 %)	7 (35 %)
Squamous cell carcinoma (*n* = 20; 29 %)	8 (40 %)	12 (60 %)	2 (10 %)	3 (15 %)	7 (35 %)
Large cell carcinoma (*n* = 5; 7 %)	4 (80 %)	1 (20 %)	–	–	1 (20 %)
NSCLC NOS (*n* = 2; 2 %)	1 (50 %)	1 (50 %)	–	1 (50 %)	–
Disease stage of all patients					
Early stages IIA-IIIA (*n* = 23; 33 %)	12 (52 %)	11 (48 %)	3 (13 %)	3 (13 %)	5 (22 %)
Advanced stages IIIB-IV (*n* = 47; 67 %)	27 (57 %)	20 (43 %)	5 (11 %)	3 (6 %)	12 (26 %)
Disease stage of NSCLC patients					
IIA (*n* = 4; 9 %)	2 (50 %)	2 (50 %)	1 (25 %)	1 (25 %)	–
IIB (*n* = 3; 6 %)	1 (33 %)	2 (67 %)	–	1 (33.5 %)	1 (33.5 %)
IIIA (*n* = 10; 21 %)	4 (40 %)	6 (60 %)	1 (10 %)	1 (10 %)	4 (40 %)
IIIB (*n* = 13; 28 %)	7 (54 %)	6 (46 %)	1 (7.5 %)	1 (7.5 %)	4 (31 %)
IV (*n* = 17; 36 %)	8 (47 %)	9 (53 %)	2 (12 %)	1 (6 %)	6 (35 %)
Operable tumor IIA-IIIA (*n* = 17; 36 %)	7 (41 %)	10 (59 %)	2 (15 %)	3 (18 %)	5 (29 %)
Inoperable tumor IIIB-IV (*n* = 30; 64 %)	15 (50 %)	15 (50 %)	3 (10 %)	2 (7 %)	10 (33 %)

DNA was isolated from peripheral blood serum samples. For this purpose, from each patient, 10 ml of blood have been collected into tubes covered with EDTA-K2. The samples were then immediately centrifuged at 1,400×*g* for 12 min to separate plasma from cellular components of blood. In order to remove impurities which may compromise the quality of the plasma, it was centrifuged again under the same conditions and transferred to clean tubes. The minimum volume of plasma necessary for further studies was 4 ml. Such prepared samples have been frozen in −80 °C until the time of analysis. For further analysis and to detect methylated *SEPT9* in each examined sample, we used Abbott mSEPT9 detection Kit (Abbott Molecular, USA).

To capture DNA from the studied samples, an Abbott *m*Sample Preparation System DNA kit (CE-IVD), which is based on magnetic microparticles, was used. In the first stage, DNA is captured and purified in magnetic field. The nucleic acid bound to microparticles is then eluted. Non-methylated cytosine residues are converted into uracil under the influence of disodium bisulfite. 5-methylcytosine residues remain unchanged. Bisulfite DNA modification is carried out with Abbott Bisulfite Modification Kit (CE-IVD) in T personal thermocycler (Biometra, Germany). DNA is then once again isolated and purified with Abbott *m*Sample Preparation System DNA kit and prepared for real-time PCR.

Real-time PCR was carried out on Abbott m2000rt instrument (CE-IVD). Every sample was examined in the presence of positive control, which was human plasma with addition of DNA with methylated *SEPT9* promoter (cancer cell line) and negative control (DNA with beta-actin sequences). Amplification was observed only for methylated *SEPT9* gene fragments, which were detected with probes specific to *SEPT9* promoter regions containing 5-methylcytosine. Beta-actin amplicons were created with the use of primers complementary to beta-actin sequence and were also detected with specific probes from Abbott (CE-IVD). Three real-time PCRs were carried out for each studied sample.

To evaluate the sensitivity and specificity of the *SEPT9* test, the following formulas have been used:

$${\text{Sensitivity }} = {\text{ TP}}/{\text{TP }} + {\text{ FN}}$$

$${\text{Specificity }} = {\text{ TN}}/{\text{FP }} + {\text{ TN}}$$

$${\text{PPV}} = {\text{TP}}/{\text{TP }} + {\text{ FP}}$$

$${\text{NPV}} = {\text{TN}}/{\text{TN }} + {\text{ FN}}$$

TP—True-Positive results, FN—False-Negative results, FP—False-Positive result, TN—True-Negative results, PPV—Positive Predictive Value, NPV—Negative Predictive Value.

Additionally in our study, we estimated concentration of free circulating DNA in plasma of lung cancer patients and in healthy individuals group. Concentration of plasma DNA was measured using BioPhotometer plus spectrophotometer (Eppendorf, Germany). The data are presented as the mean and median ± standard deviation. Mann–Whitney *U* test was used to show statistical significance of differences between studied groups. Chi-square test was used to compare the quantity of patients with different status of *SEPT9* methylation. A level of *p* < 0.05 was assigned significant.

## Results

The results of *SEPT9* promoter methylation analysis had a qualitative character (positive or negative) due to the fact that the test has CE-IVD certificate. Detection of beta-actin DNA amplification (negative control) allowed to verify an endogenous sample in respect to properly conducted DNA isolation, modification with bisulfite and real-time PCR parameters. For each examined sample (lung cancer patients and healthy individuals), beta-actin amplification was obtained.

**Fig. 1:**
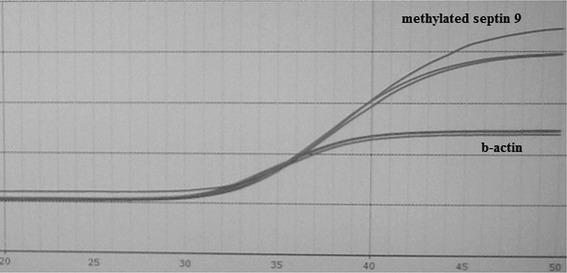
The representative positive result of septin 9 methylation examination

**Fig. 2:**
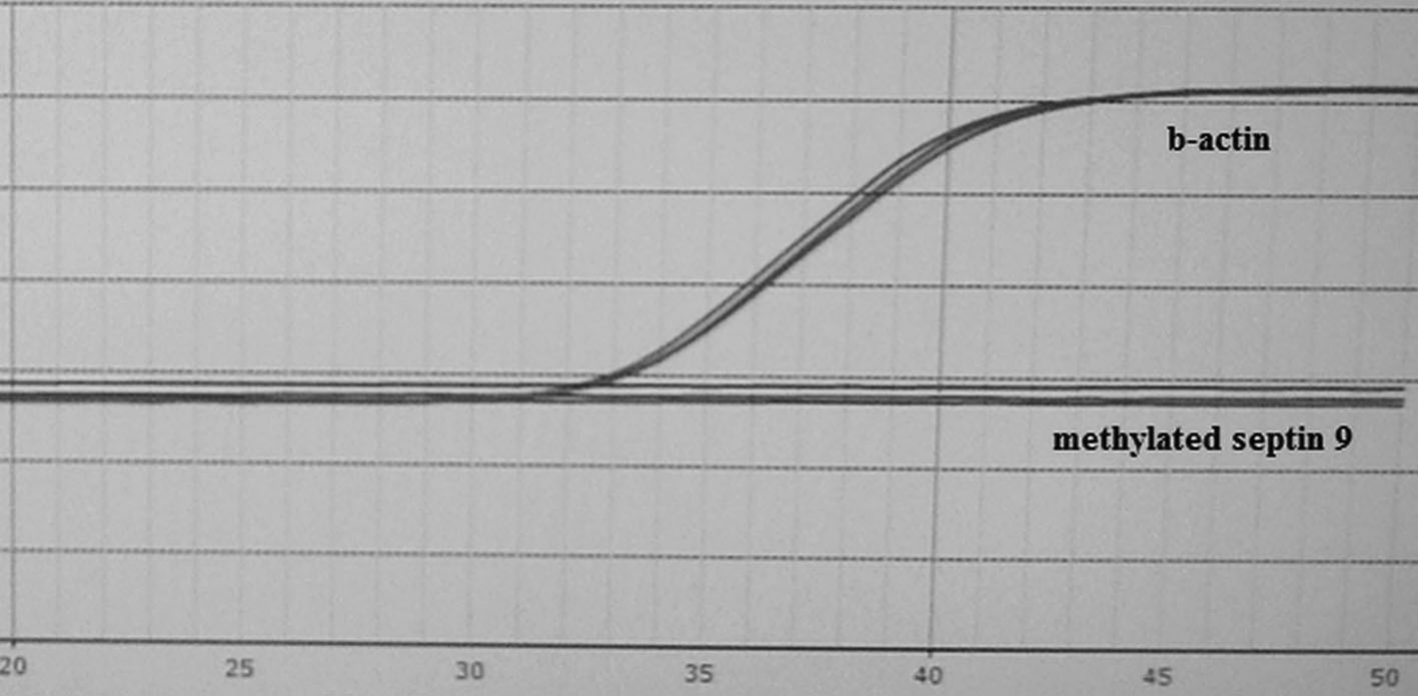
The representative negative result of septin 9 methylation examination

The examined sample was considered positive when a negative control probe signal was valid, and a positive signal from specific probe for methylated *SEPT9* promoter was detected (presence of amplification product of methylated *SEPT9* promoter region) in one out of three repeats of the same sample. Positive sample result of cancer patient was showed on Fig. 1. The sample was considered negative when, in every repetition of the sample, a beta-actin signal was valid, and no signal for methylated *SEPT9* promoter was detected (Fig. 2).

The presence of methylated *SEPT9* promoter region amplification was observed in 31 (44.3 % of the studied group) lung cancer patients (53 % of the NSCLC patients and 26 % of the SCLC patients), in a similar proportion in both genders. We detected product of amplification in real-time PCR assay in 10 patients (14.3 %) in 1 out of 3 replicates of the sample and in 8 patients (11.3 %) in 2 out of 3 replicates. However, in 16 positive patients (26 %), methylated *SEPT9* was detected in all replicates of examined samples (3 out of 3). In 4 healthy individuals (4 %), *SEPT9* methylation was detected in 1 out of 3 replicates of examined samples.

In 10 lung cancer patients and 4 healthy individuals, among whom *SEPT9* methylation have been detected in 1 out of 3 replicates, the determination was repeated in order to reject false-positive results. Methylation was detected in 7 NSCLC patients (10 %) in 1 out of 3 replicates and in 2 patients in 2 out of 3 replicates. One NSCLC patient and all healthy individuals were found to be negative, as in repetition of the determination, no PCR products have been found in any of 3 replicates (false-positive results). Furthermore, among all healthy individuals, clinical tests did not confirm any malignancies.

**Fig. 3:**
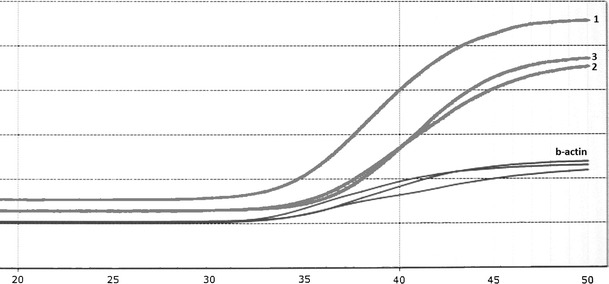
Amplification curves for control DNA with septin 9 methylation diluted with unmethylated DNA: *1*-undiluted DNA, *2*-control DNA diluted twofold, *3*-control DNA diluted fivefold

**Fig. 4:**
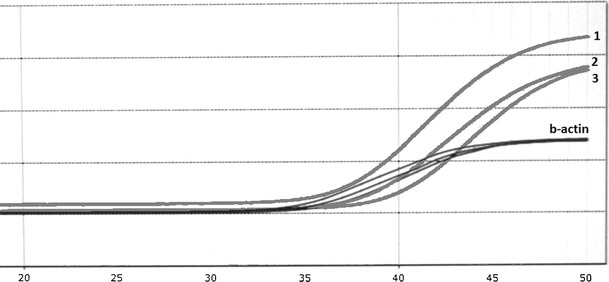
Amplification curves for control DNA with septin 9 methylation diluted with unmethylated DNA: *1*-control DNA diluted 10-fold, *2*-control DNA diluted 20-fold, *3*-control DNA diluted 100-fold

Based on these results, specificity, sensitivity, PPV and NPV were determined. Sensitivity was 44.3 %, specificity was 92.3 %, PPV—91.2 % and NPV—71.1 %. In addition, sensitivity of *SEPT9* methylation determination method was estimated. For this purpose, methylation was determined in control DNA with methylated promoter region and excluded *SEPT9* methylation that was diluted 2, 5, 10, 20 and 100-fold. In all dilutions, PCR product was detected, wherein number of cycles after which amplification was observed depended on dilution of control DNA (Figs. 3, 4).

The genetic marker was present in patients with different pathological diagnosis and in different disease stage, also in early IIA and IIB stages. Positive result of the *SEPT9* methylation was observed more frequently in squamous cell carcinoma (60 %) than in SCLC patients (26 %) and significantly more frequently in NSCL than SCLC patients (*p* = 0.032). Using chi-square test, the differences between numbers of patients with various status of mS9 were not shown. However, it is worth mentioning that the determination of *SEPT9* promoter methylation frequency in subgroups with different pathological diagnosis is unreliable because of its low number (Table 1).

**Fig. 5:**
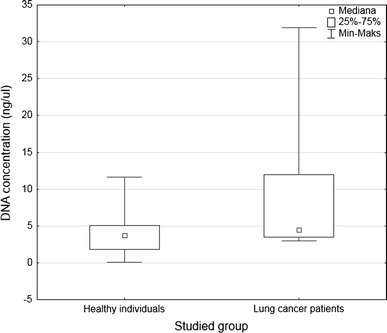
Differences in fc-DNA concentration between lung cancer patients and healthy donors

The values of plasma DNA concentration ranged from 2.5 up to 31.9 ng/μl (Mean 8.56 ng/μl) in non-treated lung cancer patients and from 0.1 up to 17.9 ng/μl (Mean 3.92 ng/μl) in healthy individuals group. Lung cancer patients showed significantly higher concentration of free circulating DNA (Median 4.56 ng/μl) than healthy group (Median 3.19 ng/μl, *p* = 0.0006) (Fig. 5).

## Discussion

Currently, lung cancer is mostly detected in advanced stage which excludes surgery and thus full recovery. The reason for such phenomenon is an insidious development of the disease, without characteristic symptoms, and its underestimation by patients. Patients’ fear of cancer and lack of disease suspicion by first contact physicians, despite first symptoms, have also significant role in late diagnosis. Therefore, it is crucial to develop easy to access, simple in execution, and fast and sensitive molecular biology techniques, which would allow early lung cancer diagnosis. Preventive use of CT or PET-CT dose not meet these criteria (especially in the matter of availability), and regular chest X-rays have too low sensitivity. Moreover, molecular test may be useful in whole population screening, and their results could qualify selected patients to more sensitive imaging examinations. Doing so would increase effectiveness of prophylactics and would rationalize its costs [1–3].

Biochemical cancer markers such as carcino-embryonic antigen (CEA) are not specific and have low sensitivity in early detection of lung malignancies; hence, they have only limited usefulness in monitoring and evaluation of the disease after performing surgery or chemotherapy. Due to the ability of detection of epigenetic changes in fc-DNA in peripheral blood, molecular markers appear to be ideal candidates for early and noninvasive diagnostics of cancer. Increased number of methylated *SEPT9* promoter region DNA sequences was observed in colorectal and rectal cancer patients in comparison with healthy individuals (among whom methylation level was below the threshold of sensitivity of molecular methods). Such a phenomenon was used in detection of colorectal cancer.

Usefulness of *SEPT9* promoter methylation examination was evaluated in a prospective clinical trial PRESEPT. It concerned early screening of colorectal cancer. Almost 8,000 individuals (over 50 years of age), without disease symptoms, with indication for colonoscopy, were qualified. *SEPT9* gene promoter methylation in fc-DNA from venous peripheral blood was found in 67 % of patients with confirmed (by colonoscopy) colorectal cancer [6]. Similar results were obtained by Grützmann et al. [7] who detected *SEPT9* promoter methylation in peripheral blood in 73 of 126 colorectal cancer patients (58 % of the studied group) in the first test study. Sensitivity of the used test depended on disease stage. Positive result was obtained in 50 % of patients in early stage of disease and in almost 100 % of patients in advanced stage of colorectal cancer.

Studying promoter methylation of *SEPT9* gene in peripheral blood plasma is a multi-stage process, involving DNA isolation, purifying and elution of the isolated nucleic acid and its modification with bisulfite, and in the end real-time PCR. In order to improve sensitivity and specificity, a validation of *SEPT9* promoter methylation detection method and optimization of its each stage were proposed. Some elements, such as application of an internal control and successive dilution of the sample prior to the assay, have been added. These changes contributed to raise detection of colorectal cancer. Inclusion of an additional measurement replicate increased the sensitivity of the assay (second test study), Grützman et al. [7] detected methylated *SEPT9* promoter in plasma of 90 out of 125 patients with colorectal cancer (72 %). The same result was obtained by deVos et al. [8] who detected methylation in 65 out of 90 colorectal cancer patients (72 %). In current studies by Warren et al. and Tóth et al., molecular testing confirmed 90 and 95.6 % cases of colorectal cancer in patients with the disease confirmed with other methods. Unfortunately, the sensitivity of the molecular method in patients with stage I colorectal cancer is still low and does not exceed 56 % [5, 8]. Currently, the subject of research is the ability to detect *SEPT9* promoter methylation in other types of cancer.

Hypermethylation of *SEPT9* gene promoter was observed in head and neck, ovary and breast cancer. Although the studies were carried on tissues and cell lines, they cannot be directly applied to the results obtained in plasma. Furthermore, methylation may concern regions of *SEPT9* gene promoter other than in colorectal cancer [8]. There is also lack of reports on the evaluation of gene promoter methylation of *SEPT9* in patients with lung cancer. In only one study by Grützman et al., *SEPT9* promoter methylation was evaluated in plasma of patients with different cancer types, obtaining positive results in 11.5 % of the studied group. In this non-selected group, also 13 patients with lung cancer have been evaluated, without taking into account clinical data and pathological diagnosis, with positive result in four cases (31 % of lung cancer patients) [7].

Our results suggest usefulness of detecting epigenetic changes such as *SEPT9* promoter methylation in fc-DNA in early and noninvasive diagnosis and prevention of lung cancer. This report is one of the first in the world that concerns this problem.

It is probable that in lung cancer, similar to colorectal cancer, methylation level of *SEPT9* promoter depends on the stage of the disease [6–9], although in this study, methylation was detected in stage II as well as in stage III and IV. It is worth remembering that the studied group was small, and there were no patients included in stage I of the disease. However, a possibility to detect *SEPT9* methylation may depend on the aggressiveness of lung cancer course. There are no reports on methylation of *SEPT9* and its promoter in different pathologic types of lung cancer. Expression of *SEPT9* gene and the level of its promoter methylation may be different in small cell lung cancer and in non-small cell lung cancer, and between each subtypes. Therefore, assessment of gene promoter methylation of *SEPT9* may prove to be particularly useful in the detection of specific type or subtype of lung cancer and may be characterized with different sensitivity and specificity for each of the subtypes; although based on results from this study, it appears that positive result may be possible to achieve in every type of cancer.

Our results are similar to the first reported studies on *SEPT9* promoter methylation in colorectal cancer patients. It appears that also in this case, validation and optimization of the detection method may be the key to increase number of detected lung cancer cases, and the assessment of methylation may be a useful test in detection, monitoring and evaluation of disease and its stage, which will certainly be proven in studies carried out on larger groups of patients with colorectal cancer.
